# The potentiality of sinking EDM for micro-impressions on Ti-6Al-4V: keeping the geometrical errors (axial and radial) and other machining measures (tool erosion and work roughness) at minimum

**DOI:** 10.1038/s41598-019-52855-6

**Published:** 2019-11-20

**Authors:** Naveed Ahmed, Saqib Anwar, Kashif Ishfaq, Madiha Rafaqat, Mustafa Saleh, Shafiq Ahmad

**Affiliations:** 1 Department of Industrial and Manufacturing Engineering, University of Engineering and Technology, Lahore, Pakistan; 2 Industrial Engineering Department, College of Engineering, King Saud University, Riyadh, Saudi Arabia

**Keywords:** Biomedical engineering, Mechanical engineering

## Abstract

Ti-6Al-4V is a material of high interest in various industrial sectors including biomedical, automotive and aerospace. Conventional means of machining encounter different types of difficulties. Electric discharge machining (EDM) is not a contest of hardness. Circular impressions of micro-depth are produced in Ti-6Al-4V using four different electrode materials including aluminum, brass, graphite and copper, each assigned positive and negative polarity. In order to get precise control over the geometry of micro-impressions dimensional accuracy and tool wear must be controlled. Thus, EDM performance has been evaluated in terms of axial dimensional error (D.E_Axi), radial dimensional error (D.E_Rad), tool length reduction (TLR), and surface roughness (SR). Since the EDM process is stochastic in nature therefore in addition to tool polarity only two factors are considered as variables, i.e. discharge current and pulse-time-ratio (ration of on-time to off-time). The behaviors of each of the four electrode materials are compared together under each of the two polarities and two variables for each of the four response characteristics. The search is carried out to select the most appropriate tool electrode polarity (common for all responses) and a single common electrode capable of minimizing all the four response measures simultaneously. Moreover, microstructures of the machined impressions are discussed. Without any compromise in the minimum values of response measures, no single polarity and a single electrode are found common. However, with a slight compromise over the machining measures negative tool polarity and copper electrode served the purpose of set objectives (minimum of D.E, TLR, and SR). The expanse of compromise is found to be ≤ 50 µm in axial and radial dimensional errors, 0.8 µm in surface roughness and no compromise in tool length reduction if the copper electrode is assigned with negative polarity.

## Introduction

Machinability of Ti-6Al-4V is of immense importance due to its widespread use in many applications especially in biomedical^1^, automotive^2^, shipbuilding^3^, micro-electromechanical systems^4^ and defense industry^5^. Machinability of Ti-6Al-4V is rated as challenging^6^^,^^7^ and poor due to very low thermal conductivity, high chemical reactivity and low modulus of elasticity due to which it is considered as difficult-to-cut material and rapid tool wear is experienced during conventional machining methods^8^. Therefore, non-conventional machining processes are increasingly being employed on Ti-6Al-4V. Electric discharge machining (EDM) is among one of those non-conventional machining processes having the capability of machining difficult-to-cut materials.

The primary function of EDM is to remove the material under the action of successive electric discharges followed by the discharge frequency. The mechanism of material removal during EDM is debatable, however, the basic principles of fundamental theories have suggested that the mechanism is based on a thermal conduction phenomenon governed by heat generated from arc channels and dissipated into the tool and the work. The result is the removal of work material near the channels in the form of melting, vaporization and flushing by the dielectric^9^. Hence, the use of electric discharge machining is mainly to subtract the material from the substrate to produce 2D and 3D features. The drilling of variety of holes (small holes, blind hole, deep holes, irregular holes, and inclined holes) are possible with EDM which otherwise may be difficult by other single process especially when the target work is titanium alloy^10^. However, EDM can also be seen for purposes other than bulk material removal. Such applications of EDM include the surface texturing^11^, improvement of surface hardness of substrate through re-solidification of electrode material^12^ and surface alloying^13^. Any material demonstrating good electrical conductivity can be taken as a tool electrode in EDM. However, the materials with higher values of thermal conductivity, melting and boiling points are more suitable tool materials. High pressure and temperature are involved during material erosion due to which the tool also gets eroded. Therefore the tool material should have the acceptable mechanical strength to reduce edge weakness. Some tools remove materials efficiently but encounter great wear and some tools exhibit slight wear but erode the substrate very slowly^14^. In a review conducted by^15^, it has been reported that the most common (top five) used electrode materials in EDM are copper, graphite, copper-tungsten, brass, and tungsten carbide. They have evaluated the said electrodes used for machining metals, ceramics, and composites. The frequency and rating of electrode follow the same sequence (above said) irrespective of substrate material, i.e. copper is at the top of the list for all the substrate materials. Thus the selection of appropriate material is necessary for coping with substrate material properties. The selection of tool material may also be different for different sets of objectives as well as the optimality goal.

Finding the most suitable tool materials or alternate tool materials is the demand of modern industry especially if the substrate material is not as common as others such as titanium alloys, nickel alloys, and metal-matrix composites. In this analogy,^16^ developed copper-titanium diboride electrode material to investigate the EDM performance on monel 400 alloy. In addition to other process parameters, the percentage of titanium diboride is taken as a variable and an optimized percentage (16%) has been recommended for optimized machining results (material removal rate and tool wear). In a study presented by^17^ brass has identified as the most sustainable electrode in terms of wear when EDM of stainless steel, tungsten carbide, and aluminum is performed. Use of brass electrode lowers down the surface hardness of EDMed samples of AISI 202 stainless steel^12^. In another study, the Ti-6Al-4V electrode is used to deposit on nickel sheets to develop surface alloying. The thickness of the Ti-6Al-4V recast layer has been found in a range of 10–70 µm which improves the micro-hardness from 132.25 HV to a range of 161.61–338.25 HV^13^. The performance contest between the copper and graphite electrodes reveals that the combination of pulse on-time and off-time varies for both the electrodes for the same response characteristics such as high MRR^18^. A combination of pulse times (On and Off-time) is recommended for low tool wear during EDM of Ti-6Al-4V.

The effect of reversing the conventional polarity of the tool electrode has different effects on machining characteristics. While drilling micro-holes in 316 stainless steel negative polarity favored in high MRR and low tool wear^19^. It has been reported that the tool polarity significantly contributes (31.56%) towards MRR, oversizing and taperness of the drilled holes. Effect of tool polarity during EDM of Inconel 600 reveals that positive polarity augments the material removal rate whereas negative polarity reduces the surface roughness^20^. The shape and size of craters formed during EDM mainly drive the resulting surface roughness of the machined samples. Crater geometry also has a significant influence on other surface characteristics. Deeper and wider craters produce rough surfaces in nickel-titanium shape memory alloy. Crater size depends on discharge energy; high energy produces deep and wide craters compared to low discharge energy^21^. In a study conducted by^22^ three dimensional geometrical features of craters are discussed in micro-EDM. Furthermore, modeling of the single and overlapping crater has also been presented to better understand the erosion mechanism. In similar lines,^23^ did the finite element thermal modeling of crater formation by considering the re-solidification phenomenon that occurred inside the crater. Simulation results of their models have a close match with the experimental results with errors 10–27%. Uncontrolled sparking caused by build-up debris of tool and work materials^24^ also contributes to erosion phenomenon which eventually affects the crater geometry.

The choice of an electrode directly influences the machining efficiency and the selection of suitable electrode is, indeed of great importance. In this context, in a study^25^ the authors have evaluated the performance of tungsten, normal copper and cryogenically treated copper against material removal efficiency, crack propagation and thickness of the white layer. Cryogenically treated copper is recommended as a suitable electrode for the said machining outcomes. Tsai^9^ proposed empirical models for surface roughness using different tool materials and both the polarities and recommended that constant parametric settings and tool material cannot be suitable for multiple work materials. In their extension of the work the empirical models are developed for the material removal rate and tool wear^26^.The processing of Ti-6Al-4V through EDM is gaining researchers’ interest and the work is being carried out to study different prospects. EDM of Ti-6Al-4V performed under abrasive-mixed dielectric has shown improvements in surface characteristics especially the surface hardness of the machined samples^27^. Surface modifications in terms of improved wear resistance, surface hardness, and surface texture are frequently required especially in orthopedic implants. Such modifications have been done by^28^ on Ti-6Al-4V samples through EDM by using niobium powder mixed in the dielectric. A layer of 215 µm thickness has been coated on Ti-6Al-4V. Improvement in surface hardness and adhesion strength of the deposited layer has been evaluated. In another research^29^, the use of bioactive powder is used in the dielectric to machine orthopedic implant made of Ti-6Al-4V and material removal rate and surface roughness are evaluated. Introducing vibration in a tool or workpiece also imparts a significant impact on the EDM of Ti-6Al-4V. The results reveal that the material removal rate is better in the case of the vibration-assisted tool as compared to the unassisted tool. An improvement of 200% in machining time has been reported using a vibration-assisted electrode during deep groove machining of Ti-6Al-4V^30^. The optimum vibration frequency of 90 Hz has been recommended for better surface roughness. Micro-grooving in Ti-6Al-4V can also be seen in a study^31^ wherein the authors have employed laminated disc electrodes made of copper and tin. Micro-groove arrays with a depth of 490 µm are successfully prepared. In another study^32^ square-shaped cavities have been produced in Ti-6Al-4V through EDM using planetary tool actuation and material removal rate and surface roughness have been evaluated. Among the copper and graphite electrodes, it has been claimed that the graphite is preferable to achieve high MRR whereas copper is suitable for minimum surface roughness.

The above literature reveals that electric discharge machining of Ti-6Al-4V is being carried out in the recent years. The process is used for evaluating either the material removal at the macro-scale or improving the surface characteristics through auxiliary methods (vibration-assisted EDM and powder-mixed EDM). Very few studies are found dealing with micro-features in Ti-6Al-4V through EDM. Another highlighted observation is that the response characteristics considered under several investigations are material removal rate, tool wear rate, and surface roughness. The majority of the researchers’ interest is to achieve high MRR and surface finish. No study has been seen dealing with the dimensional accuracy of the eroded features in Ti-6Al-4V. In this research EDM of Ti-6Al-4V has been carried out to machine round-shaped micro-impressions of depth 200 µm. In order to achieve the desired machined geometries, the selection of the most suitable electrode material is the primary objective of the machinist. In this regard, the goal of the present study is to identify the most appropriate tool electrode material for machining micro-impressions in Ti-6Al-4V. Additionally, the selection of the suitable electrode polarity plays a similar role in achieving the desired feature dimensions, which is also an integral part of the set goal of the present study. The geometry of the machined impressions is evaluated in terms of axial dimensional error (D.E_Axi) and radial dimensional error (D.E_Rad). Moreover, erosion of electrode in terms of tool length reduction (TLR) and surface roughness (SR) of eroded impressions are also considered as response measures. Four different electrode materials are tested including aluminum, brass, graphite, and copper. Each electrode is assigned with positive and negative polarity to see the effect of tool polarity on the dimensional accuracy of milled impressions as well as tool wear and surface roughness. The selection of single electrode material and the preferred polarity capable of providing the optimal values of the four responses has been carried out. Taguchi L9 is used for experimentation with each electrode and each polarity. In this way, 72 experiments are performed in total. Finally, a single (most appropriate) electrode material and tool polarity commonly suitable for each of the four responses are identified capable of producing micro-impressions with minimum dimensional errors (axial and radial), minimum tool length reduction and lowest surface roughness.

## Research details

Electric discharge sinking machining on titanium alloy (Ti-6Al-4V) has been performed using four different electrode materials. The chemical composition of titanium alloy is provided in Table 1. Since the erosion in EDM involves different mechanisms mainly thermal, electrical and mechanical nature. Therefore, the properties of the tool electrode and substrate have a direct contribution to driving the machining performance. Some of the important properties (mechanical, thermal and electrical) of electrode and work materials are presented in Table 2.

**Table 1:** Chemical composition of Ti-6Al-4V.

Weight (%)	N	C	H	Fe	O	Al	V
Min.						5.5	3.5
Max	0.05	0.08	0.015	0.40	0.20	6.75	4.5

**Table 2:** Thermo-physical properties of tool and work materials.

Properties	Tool Material				Work Material	Unit
	Graphite	Copper	Aluminum	Brass	Ti-6Al-4V	
Density	1.77	8.90	2.75	8.73	4.43	g-cm^−3^
Melting Point	3300	1083	660	940	1604–1660	°C
Thermal Conductivity	400	385	205	109	6.7	W-m^−1^K^−1^
Electrical Conductivity	0.3 × 10^6^	59.6 × 10^6^	35 × 10^6^	16 × 10^6^	0.58 × 10^6^	S-m^−1^

### Framework and objectives

The selection of the most suitable electrode material concerning the substrate material is a primary concern to achieve the desired level of machining accuracy. Electrode selection doesn’t remain obvious especially when the target work material is not as common as the other materials frequently treated under EDM. For example, the process of EDM is very much matured with respect to the machining of steels, however, regarding the titanium alloys such as Ti-6Al-4V, the process is still in its exploratory stages. The reason could be the material’s properties, work-tool interaction mechanisms, the phenomenon of vaporization and expulsion of debris from the interface^33^. Aluminum, brass, graphite and copper electrodes in the form of the circular cross-section are employed in this study. Since each electrode may have different interactions and responses with the work material therefore both the polarity options (positive and negative) are considered during experimentation. Dimensional accuracy, wear of the tool, and surface integrity of the machined area are the fundamental concerns of the machinists. Thus, four performance characteristics are defined as the response measures of EDM, i.e. axial dimensional error (D.E_Axi), radial dimensional error (D.E_Rad), tool length reduction (TLR), and surface roughness (SR). With each of the four electrode materials, four machining responses are measured. The ultimate goal is to identify the most appropriate electrode and the preferred polarity having the potential of generating good machining results with minimum dimensional inaccuracies.

**Figure 1:**
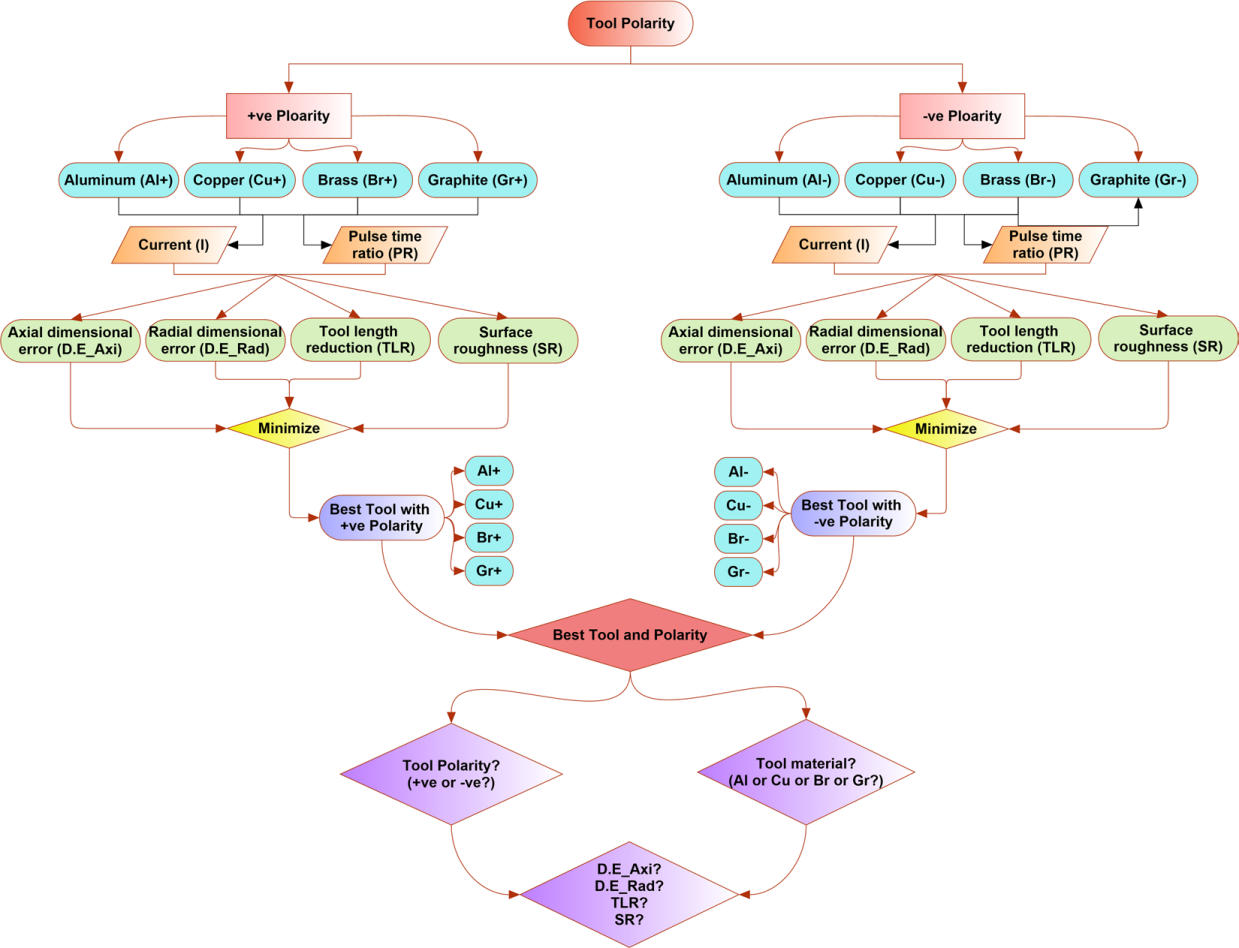
A research framework for seeking the most appropriate tool electrode and polarity for machining Ti-6Al-4V.

The detailed research framework is presented in Fig. 1. It consists of two streams of the experimentation, i.e. experiments with positive polarity and the experiments with negative polarity. Each of the four electrodes (Al, Cu, Br, and Gr) is assigned with positive polarity and the experimentation is carried out. The same experimentation is repeated with negative tool polarity. Among the EDM process parameters, the discharge current (I) and pulse time ratio (PR) are taken as the variables. Therefore, in Fig. 1 two arrows from each electrode are connected with both the variables (I and PR). Four responses named the axial dimensional error (D.E_Axi), radial dimensional error (D.E_Rad), tool length reduction (TLR), and surface roughness (SR) are the machining outputs from the two main streams of the experimentation, i.e. positive polarity and negative polarity. Since the objective is to achieve the minimum values of each of the four responses, therefore, the responses are converged through arrows towards the “minimize” objective function (diamond-shaped block in both the streams). The experimental results associated with each of the four electrodes are evaluated in terms of the four responses. At this stage, the objective was to select the best suitable tool among the four available tools. This selection of best suitable tools was categorically done in both the streams, i.e the best tool resulting in the minimum values of each response assigned with positive polarity and the best tool assigned with negative polarity. After these two selections (two best tools from both the polarities) the minimum values of the four responses are again evaluated against the set of tools. A two-way decision criterion is established, i.e. as a whole: (1) which single tool material (Al or Cu or Br or Gr) is the most appropriate for the minimum values of each of the four responses and (2) which polarity (+ve or −ve) is the most appropriate in order to achieve the minimum value of each response? Finally, under the selected tool material and the preferred polarity what are the minimum values of each of the four responses (D.E_Axi, D.E_Rad, TLR, and SR)?

Each electrode is turned to have a 20 mm diameter with a flat circular end. The length of each electrode is kept constant at 50 mm. The circular cavity with 200 µm depth is designed to achieve with maximum possible dimensional accuracy both in radial and axial directions. It is anticipated that the depth of the machined micro-impression would be less than the designed depth due to the possibility of tool wear and deposition of melt debris onto the machined surface. Another pre-envisioned assumption is that the diameter of the machined impression would be greater than the designed diameter based on the influence of plasma plume expansion in EDM along the radial direction. Therefore, the following objectives are set in this study:

*Objective 1: To minimize the axial dimensional error resulting in better accuracy along the axial direction*.

*Objective 2: To minimize the radial dimensional error resulting in better machining accuracy along the radial direction*.

*Objective 3: To minimize the tool length reduction resulting in better tool performance*.

*Objective 4: To minimize the surface roughness of the machined impression*.

*Objective 5: To select the best tool-polarity meeting all the objectives 1–4 or the maximum number of objectives from 1–4*.

*Objective 6: To select the best electrode(s) meeting all the objectives 1–4 or the maximum number of objectives from 1–4*.

### Experimentation

Experimentation has been divided into two major categories, i.e. pilot experimentation (without DOE) and mature experimentation (with DOE). During the pilot experimentation, an extensive number of trials are done to search out the suitable values of constant parameters and variables’ selection. Several parametric settings were found to be ineffective since the machining results of many of the trials were very poor. Extensive spark generation and interruption in machining have been experienced many times due to inappropriate machining parameters. Burn marks clustered at a concentrated area on the work surface are the result of excessive sparking which disrupted the machining cycle. Severity along with the evidence of such marks can be found in the upcoming section of results. The range of machine variables is set within those values ensuring the eroded surface with no evidence of burn marks. Constant parameters and variable factors are presented in Table 3. In addition to electrode polarity, discharge current (I) and pulse time ratio (PR) are considered as variable factors. In order to explore the collective contribution of pulse-on and off-time, the ratio of both of these times (pulse on-time divided by pulse off-time) is taken as a variable in this research. The levels of 0.5, 1 and 1.5 are selected in such a fashion that the pulse off-time is fixed at 50 µs and on-time is varied. Taguchi L9 orthogonal array has been selected as the design of experiment (DOE) and taken as a base-line for each electrode and polarity option. With each electrode, 9 experimental runs are executed by assigning positive polarity to the tool electrode. In this way, the first set of 36 experiments is conducted. The second set of 36 experiments is done by switching the electrode polarity from positive to negative. Thus, in-total 72 experiments are performed.

**Table 3:** List of constant parameters and variable factors with their levels.

Constant parameters			Variable Factors				
Parameter Name	Value	Unit	Factor Name	Levels			Unit
Spark voltage	5	V	Current	15	18	21	A
Spark time	4	Sec	Pulse off-time	50	50	50	µs
Flush time	4	Sec	Pulse on-time	25	50	75	µs
Arc sensitivity	3	—	Pulse time ratio	0.5	1	1.5	—
Servo sensitivity	4	—					

### Measurements and calculations

**Figure 2:**
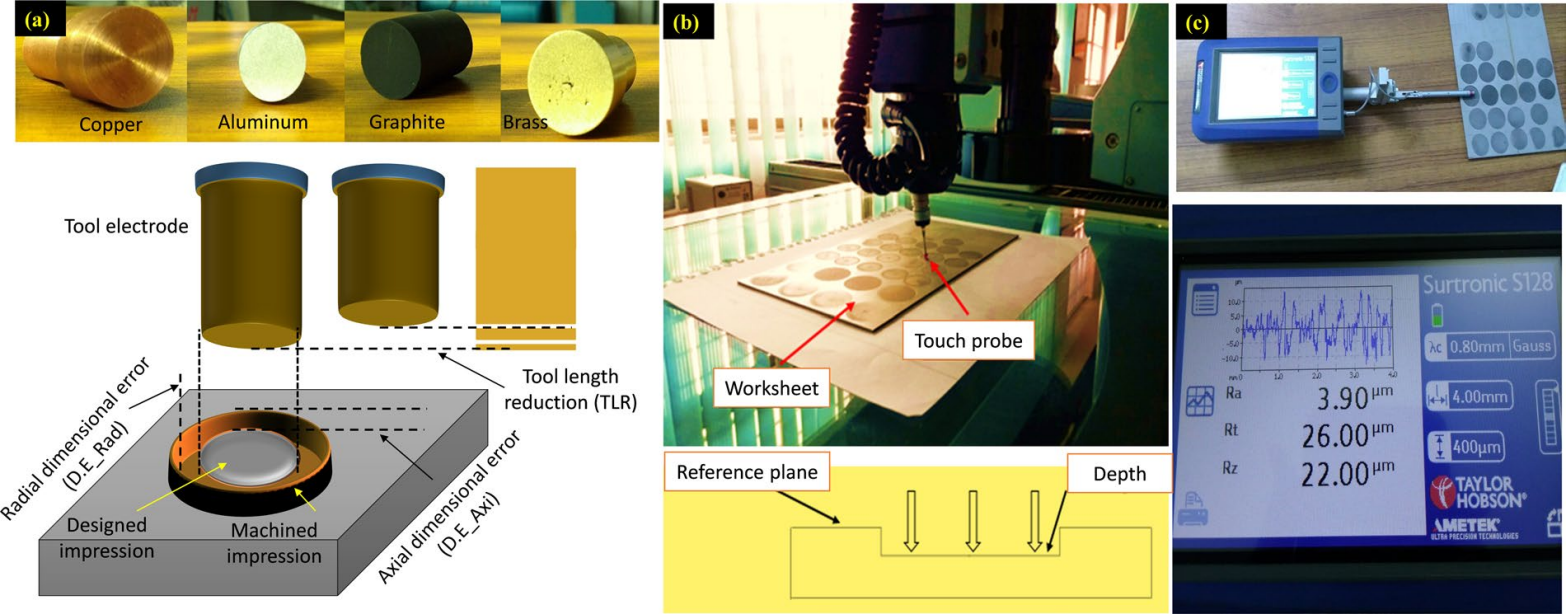
Schematics and actual measurements: (**a**) electrodes and schematic of response characteristics, (**b**) measurement of axial and radial dimensions of micro-impression through CMM, and (**c**) measurement of surface roughness.

After each experimental run, the electrode wear is observed and recorded in the form of tool length reduction (TLR) which is calculated by two methods, i.e. 1) measuring the length of electrode (through Vernier caliper) before and after machining and taking the difference, and 2) weighing the tool (through electronic weighing balance) before and after machining and converting the “difference in weight” into volume and then volume into length. Values of tool length reduction (TLR) obtained by both the methods are found precisely close to each other. Micro-depth, as well as the diameter of each of the machined impressions, have been measured through coordinate measuring machining (CMM). For the measurement of the depth of micro-impression, the surface of the un-machined region is taken as a reference plane and the machined surface is taken as a target plane. Three points on the eroded surface are marked and averaged out to gauge the actual depth of impression. A schematic is shown in Fig. 2b. In this research, a difference between the designed depth of impression (200 µm) and the actual depth of micro-impression is termed as the axial dimensional error (D.E_Axi) which needs to be minimized for an accurate machining outcome. Diametric measurements are recorded by taking a three-point measurement around the periphery of the impression. Averaged value is reported as the impression diameter. A difference between the actual radius of micro-impression and the designed radius is nominated as the radial dimensional error (D.E_Rad) which has been attempted to keep at the minimum level to ensure better geometrical accuracy. The schematic of the response measures is illustrated in Fig. 2a. The roughness of the machined surface is obtained thorough Surtronic 128 as shown in Fig. 2c. The evaluation length of 4 mm was taken for each roughness measurement. Again, in order to collect the robust measurements, surface roughness at three different points is taken and the average value is considered in the analysis. It is worth noting that three characteristics of surface roughness (R_a_, R_t_, and R_z_) were recorded but only roughness in terms of R_a_ is presented because of the wider use of this characteristic in industry.

## Results, Analysis, and Discussion

**Figure 3:**
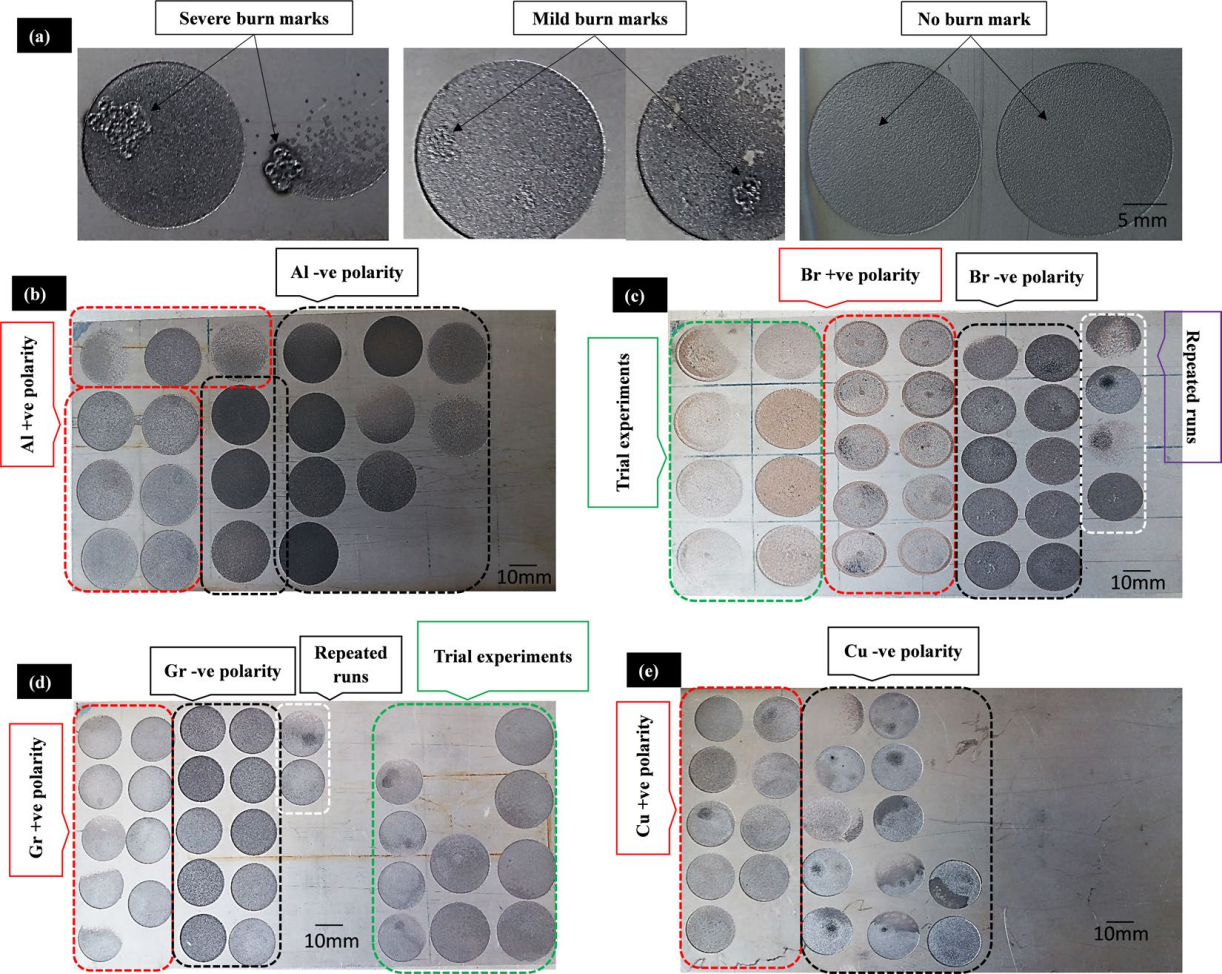
Actual machined impressions. Experimental results of: (**a**) selected pilot experimentation, (**b**) aluminum electrode, (**c**) brass electrode, (**d**) graphite electrode, and (**e**) copper electrode.

Experimental results obtained after EDM of titanium alloy are statistically, graphically and microscopically analyzed to evaluate the machining performance of each electrode material. Before performing experiments under DOE, extensive preliminary experimental trials are carried out to tune-up the constant parameters and effective range of variable factors. Inappropriate parametric values lead to poor machining results and disruption of the machining cycle. Aggressive sparking has been noticed many times due to which burn marks appeared on the machined surface as presented in Fig. 3a. Based on the severity of scald, machining is differentiated as erosion with severe burn marks, mild burn marks, and no burn marks. After getting a reasonable amount of process confidence and stability, the DOE-based experimental plan was executed. Actual machining results on Ti-6Al-4V sheets are shown in Fig. 3b–e. During the mature experimentation, some experimental runs were interrupted which were repeated later on. Callouts included as dashed-rectangles designate each set of the machined impressions, i.e. positive polarity, negative polarity, and repeated runs, etc. Four responses are considered as the performance measures namely axial dimensional error (D.E_Axi), radial dimensional error (D.E_Rad), tool length reduction (TLR) and surface roughness (SR). Experimental results (after measurements and arithmetic calculations) associated with each of the four responses are presented in Tables 4–7. The variability ( + /-) of each of the datasets associated with all the responses has also been calculated in terms of the standard deviation to see the spread of the experimental data. Each results table comprises of 8 response columns in which 4 columns represent the results under positive tool polarity and 4 columns about the results corresponding to negative tool polarity. Statistical analysis has been performed for each set of responses to get the significance and contribution of process variables. Based on a 95% confidence interval both the variables (discharge current and pulse time ratio) are found to be statistically significant so the tables related to the analysis of variance are not included in the discussion.

**Table 4:** Experimental results of axial dimensional error (D.E_Axi) during EDM of Ti-6Al-4V under different electrodes.

Exp. #	Variables	Responses								
		+ve tool polarity					−ve tool polarity			
	I	PR	D.E_Axi_Al+	D.E_Axi_Br+	D.E_Axi_Gr+	D.E_Axi_Cu+	D.E_Axi_Al−	D.E_Axi_Br−	D.E_Axi_Gr−	D.E_Axi_Cu−
**Variability** (+ **/-**)			13.77	10.13	20.90	42.57	10.51	26.28	31.01	40.69
1	15	0.5	180.00	185.67	150.33	48.67	184.00	127.67	68.50	142.00
2	15	1	147.00	169.00	136.00	136.00	161.00	134.57	11.73	86.00
3	15	1.5	156.33	183.67	125.00	139.00	178.00	147.33	37.57	130.67
4	18	0.5	170.33	173.33	149.33	168.33	189.00	106.33	88.37	100.67
5	18	1	185.67	175.67	116.53	98.67	178.67	87.00	18.10	139.00
6	18	1.5	159.00	181.00	118.33	131.67	173.67	159.67	17.40	156.33
7	21	0.5	152.67	160.67	169.57	168.67	196.33	152.33	−2.33	177.00
8	21	1	163.00	157.33	174.50	124.33	187.00	161.00	17.60	133.67
9	21	1.5	181.00	181.67	137.33	61.00	172.00	160.67	64.80	42.00

**Table 5:** Experimental results of radial dimensional error (D.E_Rad) during EDM of Ti-6Al-4V under different electrodes.

Exp. #	Variables		Responses							
			+ve tool polarity				−ve tool polarity			
	I	PR	D.E_Rad_Al+	D.E_Rad_Br+	D.E_Rad_Gr+	D.E_Rad_Cu+	D.E_Rad_Al−	D.E_Rad_Br−	D.E_Rad_Gr−	D.E_Rad_Cu−
**Variability (+/-)**			35.24	31.26	18.18	31.09	46.78	19.33	12.73	19.47
1	15	0.5	141.50	20.00	40.50	135.50	210.00	113.00	92.50	72.50
2	15	1	79.50	69.50	35.50	104.00	119.50	84.00	109.00	71.50
3	15	1.5	113.50	98.50	46.00	135.00	67.50	75.50	107.00	99.50
4	18	0.5	95.50	88.00	23.00	39.50	68.00	65.00	91.00	86.00
5	18	1	106.50	84.00	48.50	110.00	101.00	64.50	115.00	86.00
6	18	1.5	126.00	101.00	12.50	120.00	71.00	83.50	118.50	52.00
7	21	0.5	115.50	24.00	21.50	82.50	95.00	60.50	104.50	71.50
8	21	1	148.00	79.00	2.00	93.50	85.00	95.00	118.50	120.50
9	21	1.5	200.00	101.00	0.50	132.00	56.00	50.50	130.50	81.00

**Table 6:** Experimental results of tool length reduction (TLR) during EDM of Ti-6Al-4V under different electrodes.

Exp. #	Variables		Responses							
			+ve tool polarity				_ve tool polarity			
	I	PR	TLR_Al+	TLR_Br+	TLR_Gr+	TLR_Cu+	TLR_Al-	TLR_Br-	TLR_Gr-	TLR_Cu-
**Variability (+/−)**			112.75	59.37	119.66	24.19	51.16	56.32	89.75	30.24
1	15	0.5	232.03	150.19	358.99	36.29	116.02	75.09	179.50	20.00
2	15	1	232.03	112.64	358.99	72.58	116.02	112.64	179.50	36.29
3	15	1.5	232.03	75.09	179.50	108.87	116.02	75.09	179.50	72.58
4	18	0.5	348.05	150.19	179.50	108.87	116.02	225.28	358.99	72.58
5	18	1	232.03	112.64	358.99	72.58	232.03	75.09	358.99	72.58
6	18	1.5	464.06	112.64	179.50	108.87	116.02	75.09	179.50	72.58
7	21	0.5	116.02	187.73	358.99	72.58	116.02	150.19	179.50	72.58
8	21	1	116.02	262.82	538.49	72.58	116.02	37.55	358.99	108.87
9	21	1.5	348.05	75.09	358.99	72.58	232.03	75.09	179.50	72.58

**Table 7:** Experimental results of surface roughness (SR) during EDM of Ti-6Al-4V under different electrodes.

Exp. #	Variables		Responses							
			+ve tool polarity				_ve tool polarity			
	I	PR	SR_Al+	SR_Br+	SR_Gr+	SR_Cu+	SR_Al−	SR_Br−	SR_Gr−	SR_Cu−
Variability (+/−)			0.47	0.47	0.61	0.44	0.61	0.70	1.25	0.89
1	15	0.5	4.25	3.45	2.60	3.10	3.00	3.20	4.75	2.35
2	15	1	4.85	4.05	2.85	3.50	1.50	3.40	5.15	1.85
3	15	1.5	3.85	3.40	3.70	3.90	1.05	3.65	5.45	3.90
4	18	0.5	3.55	4.40	2.30	4.65	2.30	3.55	5.05	2.85
5	18	1	3.60	4.40	3.10	4.05	1.65	4.00	5.8	4.15
6	18	1.5	4.35	3.70	3.80	4.20	1.15	4.40	6.5	4.05
7	21	0.5	3.45	3.25	3.60	3.75	2.05	2.50	5.8	2.50
8	21	1	3.90	4.05	3.60	3.85	1.60	3.45	6.75	3.40
9	21	1.5	4.45	4.45	4.10	3.65	1.50	4.90	8.85	4.25

### Effect of process parameters, polarity and tool materials

The effect of process parameters (discharge current and pulse time ratio), electrode polarity (positive and negative) and electrode materials (aluminum, brass, graphite, and copper) on each of the four machining characteristics have been graphically analyzed through comparison plots. The plots are grouped into one picture in such an arrangement that the picture represents the dependence of one response over each of the process variables and electrode polarity.

#### Effect on axial dimensional error

**Figure 4:**
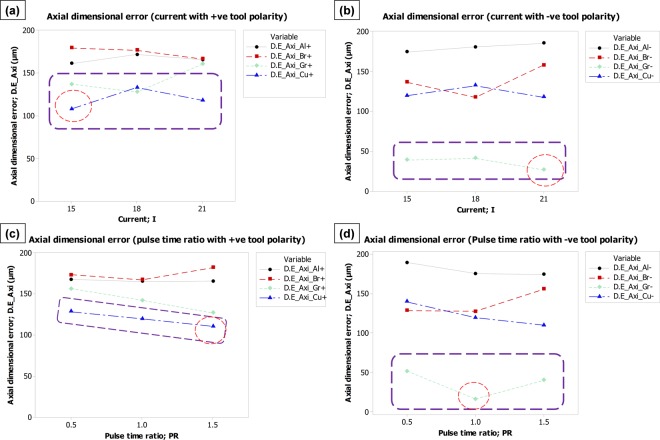
Parametric effects on axial dimensional error (D.E_Axi). Effect of: (**a**) current with +ve tool polarity, (**b**) current with −ve tool polarity, (**c**) pulse time ratio with +ve tool polarity, and (**d**) pulse time ratio with −ve tool polarity.

The effect of discharge current and pulse time ratio on axial dimensional error is presented in Fig. 4. In the case of positive tool polarity (Fig. 4a), aluminum and brass electrodes impart substantially higher axial error as compared to graphite and copper electrodes especially when the level of current is low (15 A). As the current is increased to 21 A, the error resulted from all the electrodes becomes closer to each other (∼165 µm) except for the copper electrode. The graph shows that the copper electrode gives a minimum dimensional error along the depth of impression when tool polarity is positive. However, by reversing the polarity the response of electrodes is entirely different in the context of axial dimensional error (Fig. 4b). With negative tool polarity, the mean values of axial dimensional error (D.E_Axi) caused by the graphite electrode are prominently less than the errors associated with the other three electrodes as highlighted by dashed callout. Discharge current of 21 A can give the minimum error. It is also worth noting that in case of negative tool polarity the effect of variation in discharge current is not as significant as in the case of positive tool polarity since the slopes of the lines in Fig. 4b are relatively small. The effect of pulse time ratio (PR) on axial error (D.E_Axi) is shown in Fig. 4c,d. In general, it can be stated that the increase in pulse time ratio lowers down the dimensional error. Copper electrode with positive polarity can be observed as the favorable choice to get micro-impression depth with minimum error especially at high pulse time ratio (1.5) as shown in Fig. 4c. However, the amount of error would be around 110 µm. On the other end, the graphite electrode with negative polarity occupied a distinguished position in Fig. 4d. The mean values of axial dimensional error reach to a maximum of 50 µm. Thus, comparing all the graphical facts of Fig. 4 it can be inferred that the graphite electrode and negative tool polarity could be considered as the appropriate choice with respect to axial dimensional error (D.E_Axi). Discharge current of 21 A and pulse time ratio of 1 could augment the choice of graphite for minimum D.E_Axi. Copper with negative polarity is the 2^nd^ preferred choice among tested electrodes.

#### Effect on radial dimensional error

**Figure 5:**
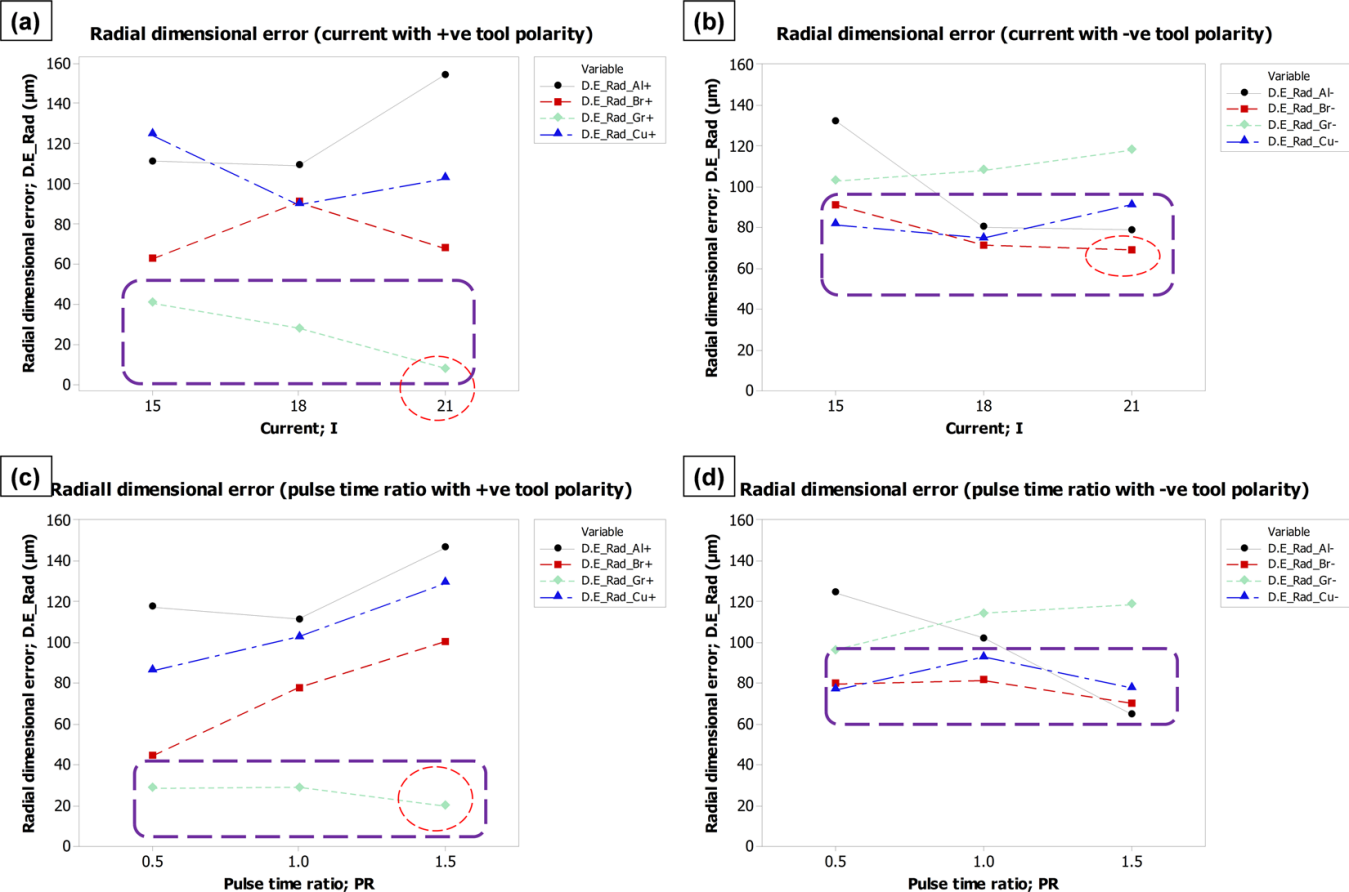
Parametric effects on radial dimensional error (D.E_Rad). Effect of: (**a**) current with +ve tool polarity, (**b**) current with −ve tool polarity, (**c**) pulse time ratio with +ve tool polarity, and (**d**) pulse time ratio with −ve tool polarity.

According to the fundamental principle of spark erosion, plasma plume is developed underneath the tool at the time of spark generation. The development of plasma allows the material to melt and generates the crater. The size of the crater mainly depends on the energy density or current density which is a function of the type of tool-work materials and process parameters. At the outer peripheral edge of the tool electrode, the plasma channel (an elliptical shape) occupies some volume beneath the tool and some volume stays outside the tool boundary. This amount of outskirt plasma causes the material to be melted from the substrate and produces a crater. The accumulation of multiple craters at the boundary of the tool and work ultimately oversized the machined feature (micro-impression in this case). In this way, the radial dimensional error came into existence. High intensity and the large volume of plasma plume permit the erosion with larger crater size. The large-sized craters eventually contribute towards the occurrence of large radial error. Figure 5 represents the effects of EDM process parameters, tool materials and polarity on radial dimensional error (D.E_Rad) experienced during EDM of Ti-6Al-4V. From the trend lines shown in Fig. 5 a&b, it can be seen that the mean effect of the discharge current on radial error follows a different trend for different materials. With the increase in current, some materials produce a larger radial error and some materials create a smaller error. The difference in these behavioral trends is mainly due to the specific tool-material properties and the resulted combination of properties along with substrate material. Extreme mismatch of thermal and electrical properties associated with tool and work may affect the electrons’ travel and finally the current density. In the case of positive tool polarity, the graphite electrode owns the lowest position in Fig. 5a and minimum radial error may be achieved at a higher current level (21 A) as marked with a red dashed circle. The amount of mean error is found to be <10 µm. On the other end with negative tool polarity and mean effects consideration, the brass electrode wins the race of minimal radial error with the discharge current of 21 A (Fig. 5b). However, it is worth noting that the minimum mean of radial error is >60 µm. By inspecting the behavior of electrodes against the pulse time ratio, three electrodes (Al, Br, and Cu) cause the high dimensional error as the pulse time ratio is increased. However, the graphite electrode exhibits a reverse trend. The mean of minimum radial error is again represented by graphite with an error of around 20 µm. The opposite result is found when the tool polarity is reversed from positive to negative (see Fig. 5d). At a high pulse time ratio, graphite produces the highest radial error. Whereas, brass and aluminum both with negative polarity have the minimum of mean radial error at pulse time ratio of 1.5. However, the minimum mean is of value >60 µm. Conclusively, it the context of achieving minimal radial dimensional error (D.E_Rad), positive tool polarity would be the preferred choice. By deciding positive polarity, the graphite electrode at higher levels of discharge current and pulse time ratio could be capable of producing a minimum radial error (Fig. 5a–c).

#### Effect on tool length reduction

**Figure 6:**
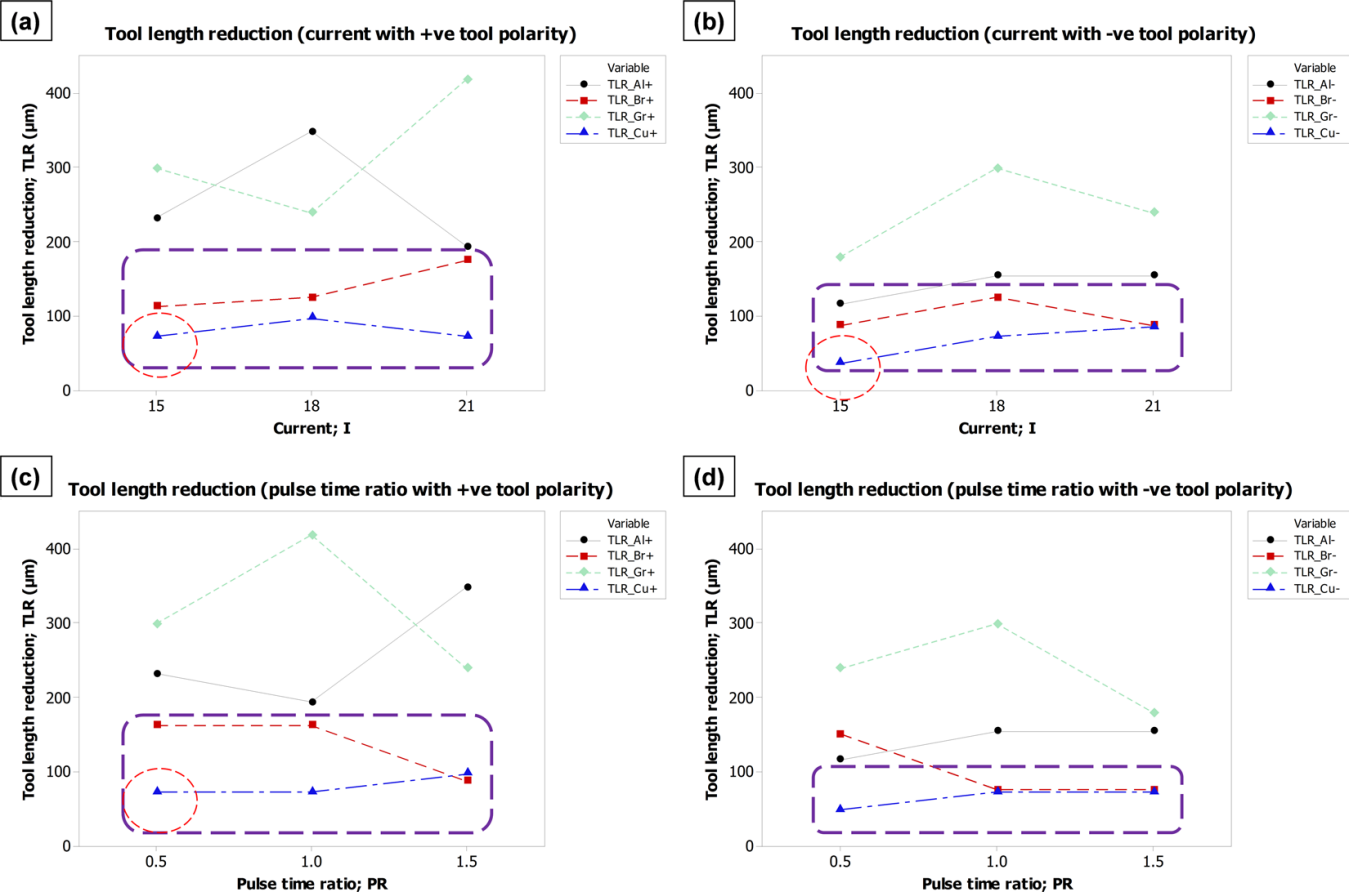
Parametric effects on tool length reduction (TLR). Effect of: (**a**) current with +ve tool polarity, (**b**) current with −ve tool polarity, (**c**) pulse time ratio with +ve tool polarity, and (**d**) pulse time ratio with −ve tool polarity.

Spark erosion is a phenomenon in which both the cathode and anode (tool and workpiece) are simultaneously eroded. However, the strength of erosion is different for the cathode in comparison to anode depending upon the assigned polarity. The erosion of the tool is the sum of the erosions in the form of vapor, liquid and solid particles as a result of the brittle rupture of the tool. Thus, the material removal from the tool, in the form of vapor and liquid, depends on the melting and boiling point of the tool material whereas the removal in the form of solid particles depends on the strength of the tool material^34^. The simultaneous melting, vaporization and particle emission of the tool electrode during each spark ultimately reduces the tool length at the end of the machining cycle. Therefore, it is essential to have control over tool length reduction during EDM. In most cases, the rate of tool erosion is kept at a low level as compared to the rate of substrate erosion. The effect of EDM parameters on tool length reduction is graphically pictured in Fig. 6. It can be observed that an increase in discharge current yields more erosion from the tool and generates a high amount of tool length reduction. A similar message can be retrieved from the trend lines against the pulse time ratio (Fig. 6c–d). Thus, it can be stated that irrespective of any tool material and polarity concern, the lower levels of discharge current (15 A) and pulse time ratio (0.5) should be selected to ensure minimum tool erosion and consequently tool length reduction (TLR). By observing the vertical-axis of each sub-graph of Fig. 6, it can be noticed that the mean value of minimum TLR is <50 µm in case of negative tool polarity whereas the graphs against positive polarity show the minimum TLR well-above than 50 µm. Thus, irrespective of electrode materials the negative tool polarity may be chosen to get better control over tool erosion. Comparing all the graphs shown in Fig. 6 and locating the lowest trend line, it can be observed that the trend line associated with the copper electrode is at the bottom of each graph. Hence, among the four tested electrodes, the copper tool could be a more promising choice in order to meet the objective related to tool length reduction (TLR). In addition to other thermal and electrical properties, material’s density also plays a significant role during EDM with respect to the melt and vaporized volume. High density means that the elemental packing factor is high and the atoms are highly compacted. The amount of melting and erosion under the same level of spark energy varies as the density of a material varies. In the case of a high-density electrode, the transmission of spark energy per atom and over the entire frontal area of the tool is low as compared to a low-density electrode. The same amount of energy has to be transferred to a relatively less number of atoms in case of low density and eventually, the tool is more aggressively gets eroded. Copper has the highest density (8.90 g-cm^−3^) as compared to the other three electrodes (see Table 2). Therefore the tool length reduction caused by the spark erosion of copper is significantly less in comparison with other electrodes (Al, Br, and Gr).

#### Effect on surface roughness

**Figure 7:**
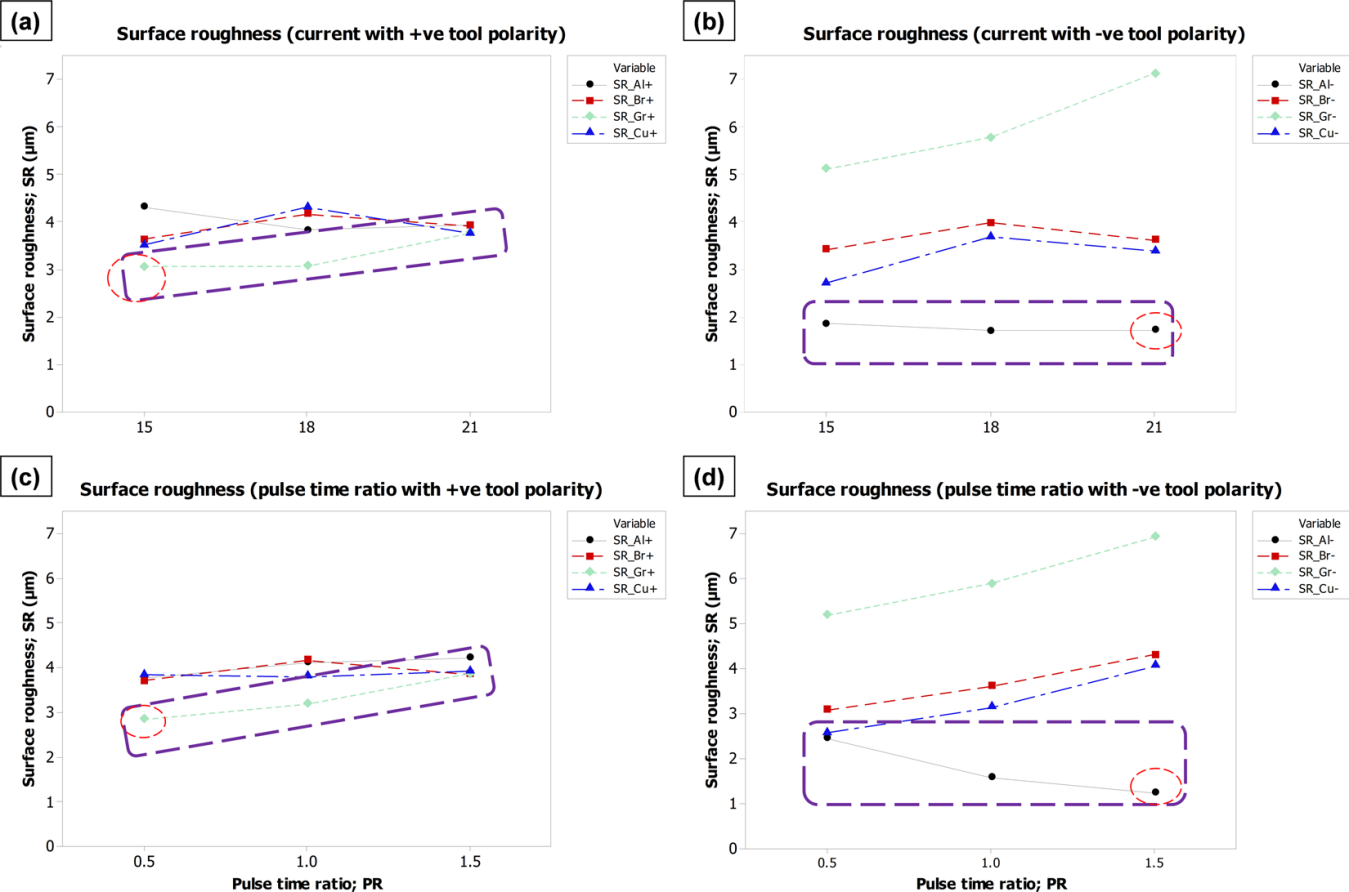
Parametric effects on surface roughness (SR). Effect of: (**a**) current with +ve tool polarity, (**b**) current with −ve tool polarity, (**c**) pulse time ratio with +ve tool polarity, and (**d**) pulse time ratio with −ve tool polarity.

Surface integrity is the function of crater size and shape. The geometry of the crater produced through EDM can be mainly described by the diameter and the depth of the crater. The overall roughness of the machined surface is the result of the overlapping of multiple craters. Shallow craters may result in better surface roughness than deep craters. Likewise, larger craters may have poor roughness than smaller craters. In addition to process parameters, the formation of crater geometry is always different for dissimilar electrode materials thus the surface roughness would, of course, be varying as it happened in case of micro-depth impressions. In addition to crater size and shape, failure to effectively evacuate the surfeited debris between the tool and the work causes the arcing phenomenon that ultimately damages the tool and work surfaces. The presence of the sedimentary debris layer in between the electrodes lowers down the breakdown strength^35^. Consequently the machining results produce variation in surface roughness. Therefore, the flushing of the minute particles (debris) is made relatively efficient by providing the large flushing time. Figure 7 depicts the mean effects of current and pulse time ratio on surface roughness (R_a_). It can be observed from the slopes of trend lines that the surface roughness of the machined impression gets coarse with the increase in discharge current (I) and pulse time ratio (PR). The y-axis scale starts from 3.0 µm and ends at 4.4 µm in case of positive tool polarity (Fig. 7a–c), whereas the scale range associated with negative polarity graphs is 1–7 µm. Hence, with respect to the effect of tool polarity on surface integrity, negative polarity may results in relatively good surface finish as compared to positive polarity. However, in the case of a positive tool polarity, the graphite electrode can produce a better surface finish with a mean value of R_a_ ~3 µm. On the other end, by reversing the tool polarity the same graphite electrode gives exceptionally coarse surface roughness as can be seen in graphs b&d of Fig. 7. Keeping the tool with negative polarity, aluminum could be the best choice since the minimum mean value of surface roughness (SR) against aluminum electrode falls around 1 µm as highlighted by rectangular and circular dashed-callouts. Another point should be noticed that lower levels of discharge current (15 A) and pulse time ratio (0.5) are required to get minimum surface roughness through positive tool polarity whereas negative tool polarity needs higher levels of discharge current (21 A) and pulse time ratio (1.5) to achieve minimum surface roughness.

### Microstructure and surface texture analysis

**Figure 8:**
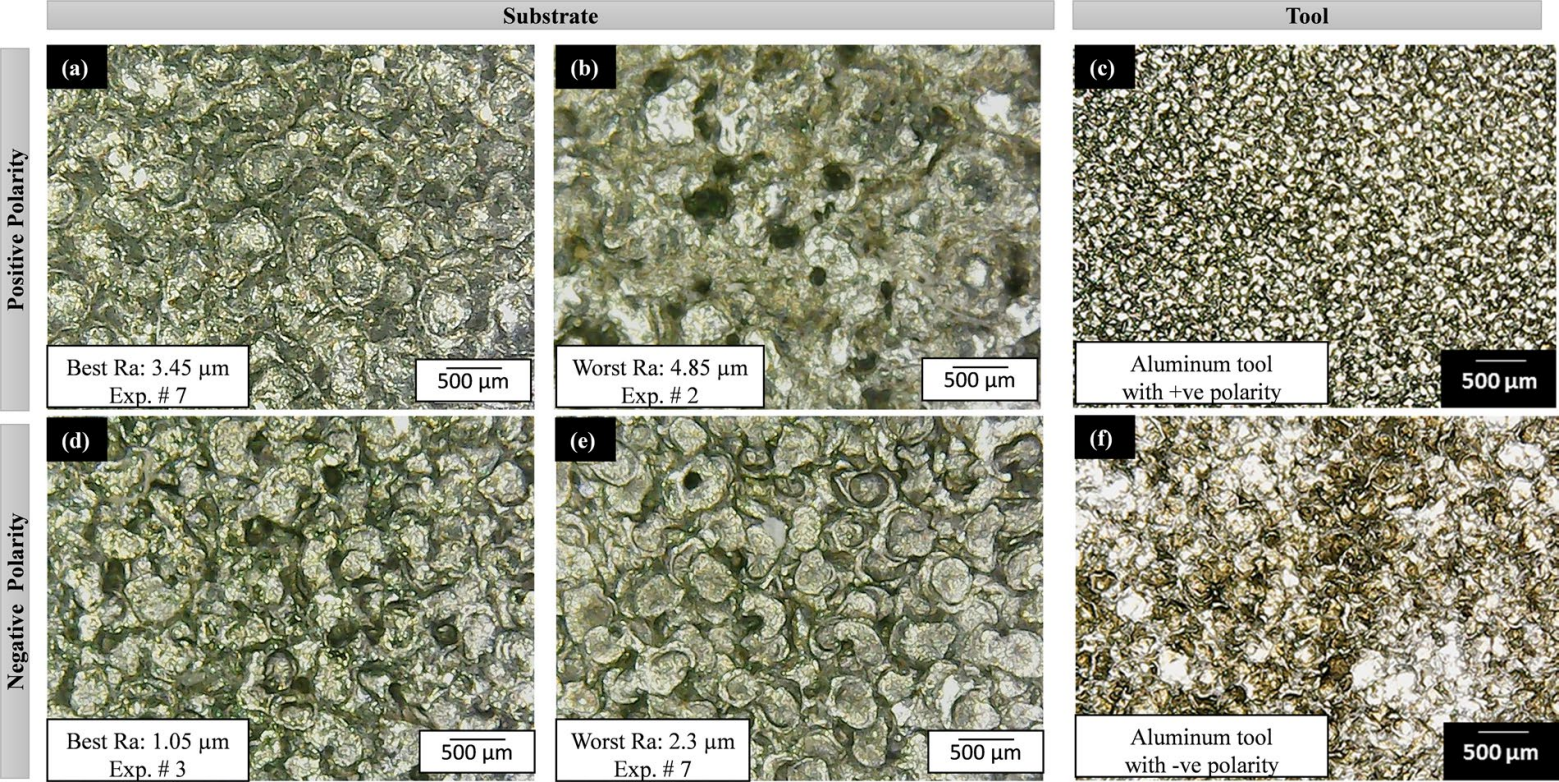
Surface texture of substrate (Ti-6Al-4V) and tool (Al) after EDM: (**a–c**) with positive tool polarity, and (**d–f**) with negative tool polarity. The selection of micrographs is based on the surface roughness criteria (best and worst) for both the polarities (+ve and −ve).

**Figure 9:**
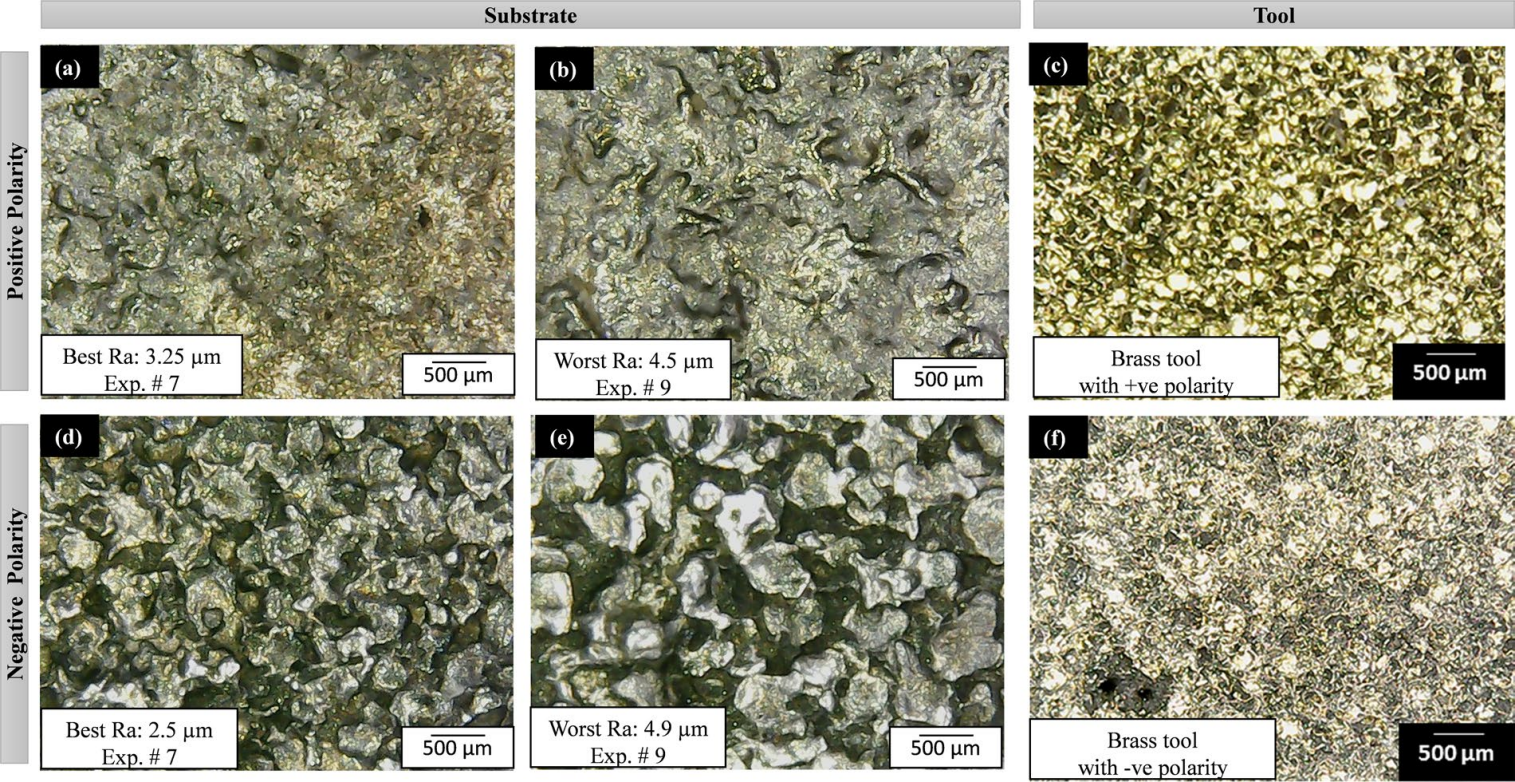
Surface texture of substrate (Ti-6Al-4V) and tool (Br) after EDM: (**a–c**) with positive tool polarity, and (**d–f**) with negative tool polarity. The selection of micrographs is based on the surface roughness criteria (best and worst) for both the polarities (+ve and −ve).

**Figure 10:**
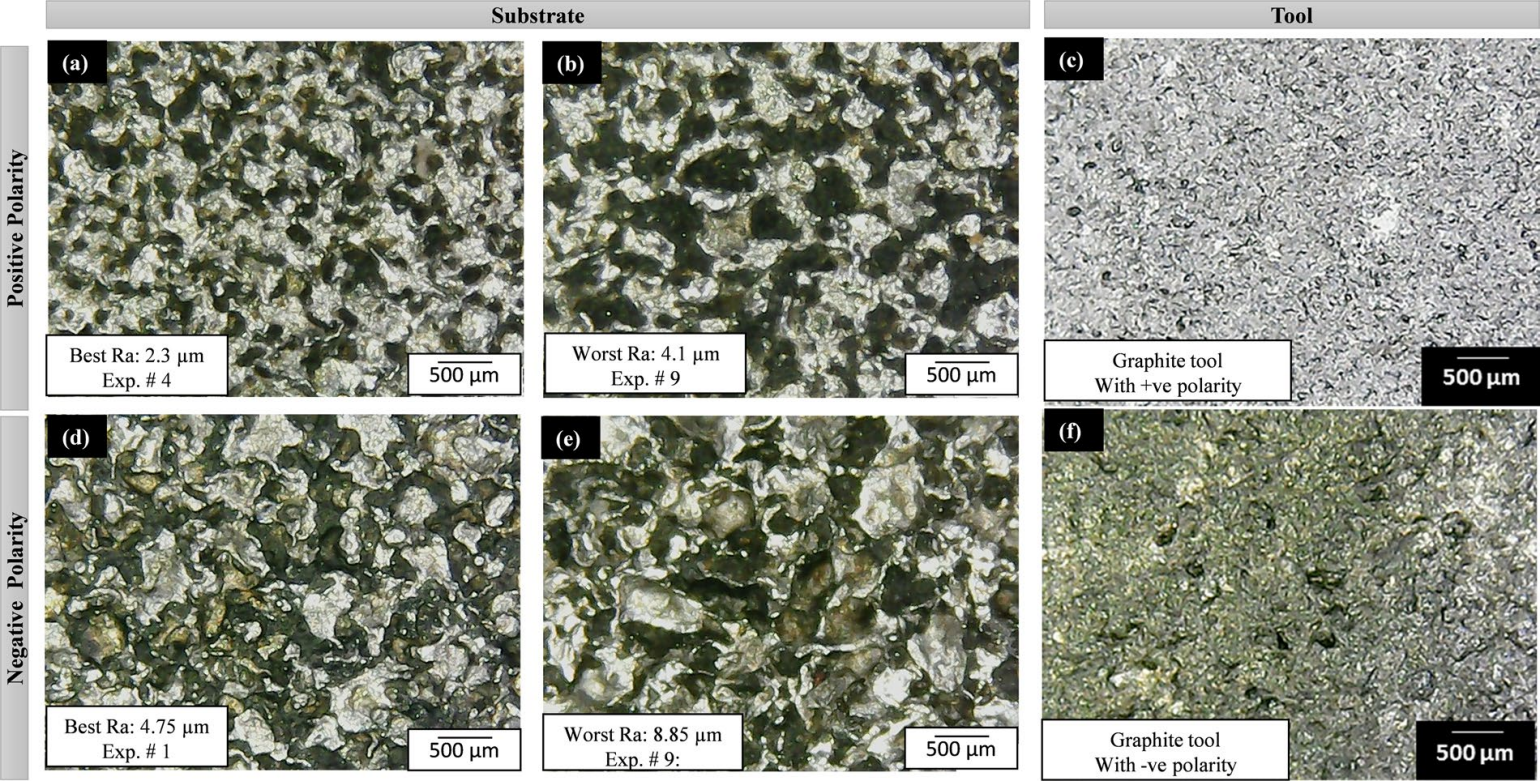
Surface texture of substrate (Ti-6Al-4V) and tool (Gr) after EDM: (**a–c**) with positive tool polarity, and (**d–f**) with negative tool polarity. The selection of micrographs is based on the surface roughness criteria (best and worst) for both the polarities (+ve and −ve).

**Figure 11:**
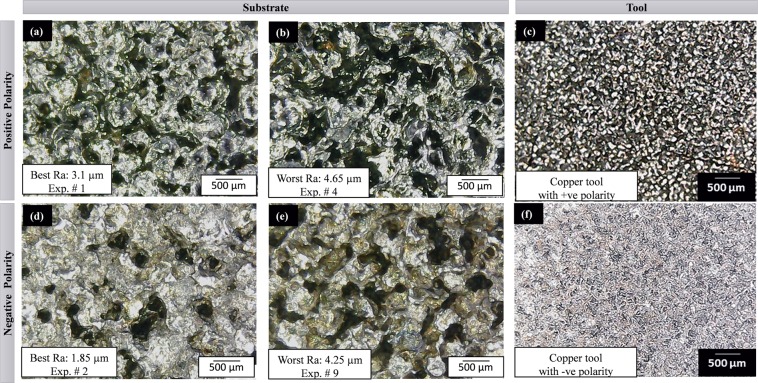
The surface texture of substrate (Ti-6Al-4V) and tool (Cu) after EDM: (**a–c**) with positive tool polarity, and (**d–f**) with negative tool polarity. The selection of micrographs is based on the surface roughness criteria (best and worst) for both the polarities (+ve and −ve).

After recording all the measurements and calculations against each of the 72 micro-impressions and 4 tools (after the experimental runs) are examined through microscopic analysis. The formation and texture of the electric discharge eroded crater may help to further augment the understanding of machining. From all the micro-graphs selected images are presented in Figs 8–11. Each figure consists of six micrographs from which four are associated with the substrate (Ti-6Al-4V) and two micro-graphs belong to the tool electrode. From a wide range of microscopic data, those microstructures of machined impressions are presented which have the best and the worst surface roughness against a particular electrode for positive polarity. The same pattern is followed by negative polarity.

The final surface texture of the machined impression is the result of crater formation and overlapping. Generally, the crater follows a circular shape viewing at the top surface. However, inside the circular shape, the molten material is ejected from the center and leaves the bowl-shaped crater. Thus, a single crater in 3D form is usually of a bowl shape. When the individual craters are overlapped due to the formation of plasma channel as well as the collapsing effect of plasma channel the overlapping of craters becomes stochastic. The formation and further collapsing of dielectric bubbles are itself stochastic and affect the uniformity of crater geometry^22^. The type of tool and work material and polarity of the electrode further contribute to enhancing this stochastic nature. Based on the observations, four types of textures or craters are identified, i.e. 1) large-sized and shallow craters, 2) small-sized and shallow craters, 3) large-sized and deep craters, and 4) small-sized and deep craters. Due to the overlapping of craters and the collapsing phenomenon of plasma channel the molten debris also gets stacked on the surface which makes the crater structure relatively spongy and final roughness of the overall machined surface gets worse. Crater size is the function of the plasma channel, i.e. enlarged plasma channel produces larger craters. It is also considered as the large-sized crater attributes towards better surface finish^27^. Care must be given to the point that the larger crater size doesn’t mean the rough surface and smaller crater doesn’t always mean the smooth surface. The shallowness and deepness of craters along with irregularly re-solidified melt debris contribute to generating rough surface texture. Based on the above discussion and by evaluating Figs 8–11, one can observe and foresee the microstructure, crater size, and texture of the EDMed impression on Ti-6Al-4V under different electrode materials (aluminum, brass, graphite, and copper).

The aluminum electrode with positive polarity (Fig. 8a,b) generates the mixed size of craters and overlapping. In some cases, it develops a spongy structure and some experimental runs show spherical texture. However, the roughness of the impression is found to be high when aluminum is considered as the anode (positive polarity) as compared to the cathode (negative polarity). It is already established that in EDM thermo-electric erosion takes place where the role of polarity is significant^36^. The surface integrity is the function of crater size and shape. The crater size is the function of melting point of the electrode. High melting point materials show trends towards smaller crater volume and relatively smooth surface^37^. The process of erosion takes place both at the electrode as well as the workpiece but which of two holds the positive polarity will be eroded more. Therefore, at positive tool polarity, the Al electrode is subjected to more erosion. When this eroded electrode comes in contact with the target surface it tends to produce the non-uniform sparking as the surface of the tool itself is irregular/spongy. This spongy nature of the tool is also evident in the micrographs provided in Fig. 8c. As the spark occurs in a non-uniform manner which causes the uneven texture at the machined area. Thus, the roughness of the machined surface is increased. Another important aspect that promotes the erosion of the tool is the smaller density (2.75 × 10^–3^ g/mm^3^) along with a low melting temperature (660 °C) of the Al electrode. The low density of the electrode is translated into loose bonding of the atoms. So, both the aspects i.e. the positive tool polarity (tool acts as the anode) and low density along with small melting temperature pronounced the erosion from the electrode surface that eventually yields poor surface finish at the target surface. Negative aluminum results in the best surface texture with R_a_ 1.05 µm. The texture of the work surface is very distinct and the crater boundaries are very much prominent (Fig. 8d). In this case, the crater size is small but it would be shallow as well, therefore the roughness measured is found at a minimum level. It is also noticed that the microstructure of the eroded tool is reverse in texture compared to the work surface texture. Figure 9 shows the micrographs associated with the tool and substrate when brass is used as a tool electrode. With negative tool polarity, the brass generates the best surface with minimum roughness (among experimental runs performed with brass). At the same time, the tool is again of a porous-like structure (Fig. 9c). On the other end, the worst amount of surface texture (R_a_ = 4.9 µm) can be observed from Fig. 9f which is the result of the brass electrode with negative polarity. Deep craters can be observed in this micrograph. The formation of these deep craters is a result of high discharge energy produced during the EDM of the selected material. The magnitude of the discharge energy is dependent on the value of the pulse on time. In the later case, the value of pulse on-time is higher (as pulse time ratio is 1.5) which yields a greater amount of discharge energy. In the case of higher pulse on-time and shorter off-time the crater is partly smeared by the liquid phase at the rim of the crater under the action of expending discharge channel^38^. Thus, the resulting surface finish becomes poor. It has also been witnessed that the surface quality produced via the brass electrode is relatively poor in contrast to that found with the Al electrode as described in Figs 8 and 9. Craters are found at the machined surface with both the electrodes. However, the depth of the crater is pronounced in the case of brass. This is attributed to the lower thermal conductivity of the brass electrode (109 W-m^−1^K^−1^) as compared to the Al electrode (205 W-m^−1^K^−1^). The thermal conductivity of the tool material has a pivot role in determining its wear. If the thermal conductivity of the tool is lower, it means that the heat is contained at the tool interface that promotes the chances of tool wear. As the quality of the machined surface in EDM is a replica of the tool surface quality, therefore, deeper craters are found at the target surface because the tool itself contained craters owing to wear resulted from intense localized heating as depicted in Fig. 9. In the case of EDM through the graphite electrode, the microstructures of the machined impressions are relatively worst among all other textures obtained through Al, Brass, and Cu electrodes. Figure 10 depicts that there is a huge proportion of black spots within and in-between the successive craters. The presence of black spots is the indication of high carbon content present in the machined surface. The similar observation has also been reported that in the case of negative polarity graphite electrode^39^ the carbon particles ejected from the graphite gets adhered. During EDM of Ti-6Al-4V in the presence of kerosene dielectric, the TiC is formed on the work surface^40^. The highest roughness (8.85 µm) which is observed throughout the experimentation belongs to the graphite electrode with negative assigned polarity. The higher levels of current and pulse time ratio have also contributed to a high-rise in the surface texture as the crater size and depth are seeming to be of larger dimension (Fig. 10f). Therefore, the surface textures are found to be worst in the case of graphite electrode in both the cases of negative as well as positive polarity. However, the magnitude of the crater’s depth is noted higher when a negative tool polarity is employed. As described earlier the quality of the cut surface is a duplicate of surface quality of the electrode. Figure 10f demonstrates that the quality of the tool surface in observed inferior at negative tool polarity. The effect is replicated on the machined surface in the form of higher surface roughness. Another reason that contributes to the higher surface roughness of the machined area in the case of the graphite electrode is the greater magnitude of MRR associated with the use of the graphite electrode. The magnitude of MRR achieved using the graphite electrode is noticed to be highest amongst all the tool electrode materials while machining of the Ti6Al4V. This is primarily attributed to the higher melting temperature of the graphite electrode (3300 °C). In EDM the machining action is accomplished through intense heating in the workpiece-electrode gap that melts and vaporizes the workpiece surface. The material got removed both from the target surface and from the electrode. The effect of such a high heat input is more evident on that surface which has a lesser melting temperature. If the melting temperature of the electrode is higher, more of the material is removed from the work surface and vice versa. In the present case, the melting temperature of graphite is approximately double the melting temperature of the Ti-alloy (1604 °C). Thus, more the material would get removed from the target surface yielding deeper craters. Consequently, surface quality is compromised.

The microscopic analysis of machined impressions obtained after EDM with the copper electrode is presented in Fig. 11. The tool has smooth and fine surface texture when acts as the cathode and spongy texture when treated as the anode. The textures of the machined surfaces are found to be prominent when copper is on the negative side. The crater size is very large but shallow due to which the roughness is 1.85 µm R_a_ (Fig. 11d). The texture with positive tool polarity becomes more compacted but with high roughness value. A comparison of roughness values obtained using copper and brass electrode depicts that roughness magnitudes are close to each other. The main reason behind this is the similar magnitude of melting temperature of both the electrodes of materials i.e. 1083 °C for cooper and 940 °C for brass. Although the value of surface roughness is noticed similar to both the said electrode materials but in the case of cooper the magnitude of roughness is found smaller in contrast to that noted with brass. This can be explained considering the density of the cooper (8.9 g-cm^−3^). The density of the tool materials depicts the compactness of the tool and also provides an insight into the bond strength. An electrode having low density is more susceptible to wear during the EDM process and subsequently, the machined surface generated by this electrode is of inferior quality. As the density of copper is higher in a contest to brass, therefore, cooper holds lesser chances of wear which ultimately translates into a better surface quality of the machined area. It is also noticed that the graphite electrode has also been rated at the top in terms of tool length reduction (TLR) which means that graphite experiences high wear in case of machining Ti-6Al-4V.

### Screening and selection of the best possible solution

After performing the extensive set of experimentation, parametric effects and microstructural analysis, it has been observed that the selection of a unique polarity and common tool material is a very complicated assignment for a machinist. The decision becomes more complex if all the response characteristics (D.E_Axi, D.E_Rad, TLR, and SR) are simultaneously considered to keep at the desired level (minimum). A summary table has further been designed to simplify this selection process. All four responses are segregated with respect to each of the four tool materials. Only maximum and minimum response measures are extracted from the whole experimental data as shown in Table 8. Two major decision criterions are defined, i.e. 1) selection without compromise and 2) selection with compromise.

**Table 8:** Screening and selection of electrode polarity and tool material for EDM of Ti-6Al-4V.

Tool material and evaluation criterion	Sub-criterion	Responses and decisions							
		Axial dimensional error		Radial dimensional error		Tool length reduction		Surface roughness	
		D.E_Axi (µm)		D.E_Rad (µm)		TLR (µm)		SR (µm)	
		+ve	−ve	+ve	−ve	+ve	−ve	+ve	−ve
Aluminum	Max.	185.67	196.33	200	210	464.06	232.03	4.85	3
	Min.	147	161	79.5	56	116.02	116.02	3.45	1.05
	Var. (+ / −)	13.77	10.51	35.24	46.78	112.75	51.16	0.47	0.61
Brass	Max.	185.67	161	101	113	262.82	225.28	4.4	4.9
	Min.	157.33	87	20	50.5	75.09	37.55	3.25	2.5
	Var. (+/−)	10.13	26.28	31.26	19.33	59.37	56.32	0.47	0.70
Graphite	Max.	174.5	88.37	48.5	130.5	538.49	358.99	4.1	8.85
	Min.	116.53	−2.33	0.5	91	179.5	179.5	2.3	4.75
	Var. (+/−)	20.90	31.01	18.18	12.73	119.66	89.75	0.61	1.25
Copper	Max.	168.67	177	135.5	120.5	108.87	108.87	4.65	4.25
	Min.	48.67	42	39.5	52	36.29	20	3.1	1.85
	Var. (+/−)	42.57	40.69	31.09	19.47	24.19	30.24	0.44	0.89
Without compromise	No compromise on the minimal of any response								
	Response evaluation	Minimal response value among all tool materials							
		2.33 µm		0.5 µm		20 µm		1.05 µm	
	Polarity evaluation	Screening for Polarity (irrespective to tool)							
		Negative		Positive		Negative		Negative	
		Best polarity (Single): No single polarity							
		Preferred polarity (Single): Could be negative							
	Tool evaluation	Tools and their ranks for individual response							
		1. Graphite (2.33 µm)		1. Graphite (0.5 µm)		1. Copper (20 µm)		1. Aluminum (1.05 µm)	
		2. Copper (42 µm)		2. Brass (20 µm)		2. Brass (37.55 µm)		2. Copper (1.85 µm)	
		Best tool (Single): No single tool							
		Preferred tools (optional)							
		Graphite for dimensional accuracy (D.E_Axi and D.E_Rad)							
		Copper for tool length reduction and surface roughness (TLR and SR)							
With compromise	Compromise for just one response (D.E_Rad). Minimum radial dimensional error is from positive polarity. If we take minimum of D.E_Rad from negative polarity then….								
	Response evaluation	Minimal response value among all tool materials							
		2.33 µm		50.5 µm		20 µm		1.05 µm	
	Polarity evaluation	Screening for Polarity (irrespective to tool)							
		Negative		Negative		Negative		Negative	
		Best polarity (Single): Negative							
	Tool evaluation	Tools and their ranks for individual response							
		1. Graphite (2.33 µm)		1. Brass (50.5 µm)		1. Copper (20 µm)		1. Aluminum (1.05 µm)	
		2. Copper (42 µm)		2. Copper (52 µm)		2. Brass (37.55 µm)		2. Copper (1.85 µm)	
		Best tool (Single): Copper							
Expanse of compromise	Differences between actual minimum responses values and compromised response values								
		D.E_Axi: 41.77 µm		D.E_Rad: 50 µm		TLR: 0 µm		SR: 0.8 µm	

The criterion without compromise means that the selection of tool polarity and tool material is entirely based on minimum response values (which is the primary concern). A minimum value of each response is identified without giving any importance to polarity and tool material. Then polarity evaluation is carried out in which tool polarity associated with each response is adopted returning into a minimum measure of a particular response. In this way, negative tool polarity is found for axial dimensional error (D.E_Axi), tool length reduction (TLR) and surface roughness (SR). Radial dimensional error is found to be at the lowest value with positive tool polarity. Hence, a single common polarity suitable for all the four responses is not achieved if no compromise criterion is truly followed. However, negative polarity can be preferred since it conforms to 3 out of 4 responses. Now evaluating the tool materials, there is no single tool that can result in minimum measures of the complete set of responses. In order to make the tool evaluation more rigorous, tools are ranked against each response. The first rank is given to the tool yielding 1^st^ minimum measure and the second rank is assigned to the tool resulting in 2^nd^ minimum of the same response. Looking into the options of 2^nd^ ranked tools corresponding to each response, again no common tool is observed. However, by sticking on the “no compromise criterion”, the choice of the preferred tools has been extracted. Graphite tool is truly suitable for minimum dimensional errors (D.E_Axi and D.E_Rad), the copper tool can be preferred with respect to minimum tool length reduction (TLR) and the aluminum tool is the most appropriate for creating lowest surface roughness. Conclusively, it can be stated that single tool material with common polarity is not possible if each response measure is not compromised in any sense.

On the other end, a second decision criterion is established named as a selection with compromise. The same procedure is followed except compromising one response, i.e. radial dimensional error (D.E_Rad). In actual, the minimum radial error (of amount 0.5 µm) is achieved with positive tool polarity. If we consider a minimum of radial error from the negative polarity data pool, then the problem of common polarity can be resolved. In this way, negative polarity can be assigned for all the four machining responses just with a slight compromise to D.E_Rad. Next is to identify a single common tool material. In a similar fashion as explained earlier, two tools per response characteristic are taken at a time and are ranked further. In this way, the copper electrode has qualified as a single common electrode meeting all four responses (D.E_Axi, D.E_Rad, TLR, and SR). In must be noted that, the set of tool materials all having 1^st^ rank doesn’t contain a single common tool. Therefore, for TLR copper is picked from the 1^st^ ranked tools whereas for D.E_Axi, D.E_Rad, and SR the copper is grabbed from the 2^nd^ ranked tools. Conclusively, it can be stated that with a slight compromise on radial dimensional error and ranking of tools, the copper electrode can serve the purpose of electric discharge machining of Ti-6Al-4V with control over dimensional errors (D.E_Axi and D.E_Rad), tool erosion (TLR) and surface roughness (SR). With the above-said compromise, a common tool polarity (negative) can also be decided for each response.

After screening and selection of tool polarity as well as tool materials, the amount of compromise has also been evaluated for each response. Minimum response measures from the list of “without compromise criterion” are taken as benchmark values. This list comprises of a minimum of responses with values: 2.33 µm D.E_Axi, 0.5 µm D.E_Rad, 20 µm TLR, and 1.05 µm SR. On the other end, the values from the list of “with compromise criterion” consists of: 42 µm D.E_axi, 52 µm D.E_Rad, 20 µm TLR, and 1.85 µm SR. Differences between the benchmarked values from “without compromise criterion” and values from “with compromise criterion” are calculated. These differences corresponding to each of the four responses are provided in the last row of Table 8. Hence, the expanse of compromise consists of 41.77 µm in axial dimensional error (D.E_Axi), 50 µm in radial dimensional error (D.E_Rad), 0 µm in tool length reduction (TLR) and 0.8 µm in surface roughness (SR).

Hence it can be inferred from the above description that no single electrode material is found to be suitable for producing the minimum value of each of the four responses (D.E_Axi, D.E_Rad, TLR, and SR) while EDM of Ti6Al-4V. Similarly, the minimum values of the set responses, considered simultaneously, cannot be achieved using a single polarity. Single electrode and single polarity are not found common to achieve minimum values of the responses. However, by a slight compromise in the minimums of the responses, one electrode and one tool polarity can easily be filtered out. In this case the copper electrode with negative polarity is found to be suitable and the expanse of compromise in the minimum values of the responses is 41.77 µm in axial dimensional error (D.E_Axi), 50 µm in radial dimensional error (D.E_Rad), 0 µm in tool length reduction (TLR) and 0.8 µm in surface roughness (SR). These founds of the present research can be considered as significant contribution in the existing literature.

## Conclusions

Electric discharge machining of titanium alloy (Ti-6Al-4V) has been carried out with four electrode materials (aluminum, brass, graphite, and copper) using positive and negative tool polarities. Round-shaped micro-impressions (200 µm designed depth) are produced. The goal is to identify a single electrode with common polarity capable of producing micro-impressions with minimum amounts of axial dimensional error (D.E_Axi), radial dimensional error (D.E_Rad), tool length reduction (TLR), and surface roughness (SR). Based on the experimental data, parametric effects, microstructures of the eroded impressions, polarity effects and tool materials effects, following sound conclusions may be drawn:

1. Electric discharge machining of Ti-6Al-4V with precise control over individual response characteristics is easy to maintain. However, simultaneous control over all four performance characteristics (D.E_Axi, D.E_Rad, TLR, and SR) is highly difficult to achieve.
2. For most of the responses, positive tool polarity results in aggressive machining errors. However, negative tool polarity can be set to achieve relatively accurate machining results with a slight negotiation for radial dimensional error (D.E_Rad).
3. With no compromise on minimum response measures, different electrodes and different tool polarities are required to be selected. Negative graphite gives a minimum of 2.33 µm D.E_Axi, positive graphite allows a minimum of 0.5 µm D.E_axial, negative copper yields lowest tool length reduction (TLR) of 20 µm, and a negative aluminum electrode is the best for achieving minimum surface roughness (SR) of micro-impression (R_a_ = 1.05 µm).
4. No single electrode material (among the four tools employed; Al, Br, Gr, Cu) is capable of producing micro-impressions without compromising response characteristics.
5. Copper electrode assigned with negative polarity could be considered as the most appropriate and single choice for EDM of Ti-6Al-4V meeting the requirements of all the four performance measures (D.E_Axi, D.E_Rad, TLR, and SR). However, it results in the performance measures with values slightly above the minimum values achievable through different electrodes and polarities.
6. A distinct expanse of compromise in EDM performance measures accounts for an increase in the values with an amount equals to: 41.77 µm in axial dimensional error (D.E_Axi), 50 µm in radial dimensional error (D.E_Rad), 0 µm in tool length reduction (TLR) and 0.8 µm in surface roughness (SR).
7. Microscopic analysis reveals that the surface texture of the machined impressions corresponding to graphite and copper electrodes contain a relatively high proportion of black spots (probably carbon content). Moreover, graphite creates the coarsest surface among four electrodes (R_a_ = 8.85 µm). The craters produced through the aluminum electrode are more uniform and have clear boundaries. That is why the impressions machined with aluminum electrode have the lowest value of surface roughness (R_a_ = 1.05 µm).
8. With respect to parametric effects, each electrode behaves differently under each of the two polarities (+ve and −ve). The influence of discharge current and pulse time ratio (ratio of pulse on-time and off-time) further varies with the change in tool materials against each of the four responses. Generally, with the increase in discharge current and pulse time ratio the machining errors are increased. However, future research to optimize the process parameters, as well as an additional study of machined surface textures, is also further needed which would expectedly be the forthcoming extension of this work.
